# Pyrroloquinoline quinone promotes porcine oocyte *in vitro* maturation and subsequent embryo development by enhancing lipid metabolism and improving mitochondrial function

**DOI:** 10.5713/ab.24.0847

**Published:** 2025-04-11

**Authors:** Zehua Zhang, Zhigang Gao, Zhenwei Jia

**Affiliations:** 1 College of Animal Science and Technology, Inner Mongolia Minzu University, Tongliao, China

**Keywords:** Embryo Development, Lipid Metabolism, Mitochondria Function, Oocyte *In vitro* Maturation, Pyrroloquinoline Quinone

## Abstract

**Objective:**

The present study evaluated the beneficial effects of pyrroloquinoline quinone (PQQ) on *in vitro* maturation (IVM) of porcine oocyte and subsequent early embryo development.

**Methods:**

Porcine cumulus oocyte complexes were cultured in IVM medium with supplementation of 0, 200, 400, 800 or 1600 nM PQQ for 42 h. We first examined cumulus expansion index (CEI) and the rate of oocyte nuclear maturation. Then, we assessed oocyte mitochondrial function, oxidative stress levels, lipid metabolism and subsequent embryonic development.

**Results:**

PQQ (800 nM) supplementation signiﬁcantly increased CEI and the nuclear maturation rate of oocytes following IVM. Additionally, oocysts supplemented with 800 nM of PQQ showed signiﬁcantly increased mitochondrial content, mitochondrial membrane potential, activity, and mRNA expression levels of genes associated with mitochondrial biogenesis (*PGC-1a, NRF1, NRF2* and *TFAM*). PQQ signiﬁcantly reduced the levels of reactive oxygen species, lipid drop-lets, and fatty acids content, while enhancing the mRNA expression levels of genes related to antioxidant activity (*SOD1, SOD2, GPX* and *CAT*), lipolysis (*ATGL* and *HSL*) and β-oxidation (*CPT1B* and *CPT2*) in porcine oocytes. PQQ (800 nM) supplementation signiﬁcantly increased cleavage rate, blastocyst formation rate, and total blastocyst cell numbers following partheno-genetic activation.

**Conclusion:**

PQQ supplementation during IVM positively influences porcine oocyte maturation and subse-quent embryonic development by enhancing mitochondrial function and lipid metabolism and alleviating oxidative stress.

## INTRODUCTION

Oocyte *in vitro* maturation (IVM) is an important technology to generate mature oocytes and embryos for human infertility treatment and animal reproduction. However, it is generally accepted that the quality and developmental potential of IVM oocytes is lower than those of their *in vivo* counterparts, probably partly due to insufficient cytoplasmic maturity [1]. Mitochondria are the most abundant organelles in cells, and they are thought to play important roles in determining oocyte cytoplasmic maturation [2]. It is well established that mitochondria are not only the cells’ energy generators, but also the major sites of reactive oxygen species (ROS) production. Nevertheless, excessive accumulation of ROS can cause oxidative stress, which may subsequently lead to mitochondrial dysfunction, decreasing the developmental competence of *in vitro* maturated oocyte [3]. Therefore, mitigating oxidative stress and strengthening mitochondria function is an effective strategy for improving the developmental competence of *in vitro* matured oocyte.

It is generally known that porcine oocytes contain a high content of intracellular lipids that are primarily stored in lipid droplets as triglycerides. Endogenous triglycerides are integral for energy metabolism during porcine oocyte IVM. However, the excessive lipid accumulation in oocytes is harmful to oocyte development *in vitro* [4]. Lipid metabolism via the fatty acid oxidation pathway produces adenosine triphosphate in mitochondria, which is important for oocyte maturation and embryo development [5]. Thus, regulation of lipid metabolism is beneficial for promoting mammalian oocytes IVM and embryo development [6].

Pyrroloquinoline quinone (PQQ), a water soluble, thermo-stable triglyceride-quinone, is identified initially as a mammalian vitamin-like redox cofactor [7]. It has been shown that PQQ is important for mammalian growth, development, reproduction and immune function [8]. PQQ can promote the expression of PGC-1α, which is a critical factor in regulating mitochondrial biogenesis and oxidative metabolism [9]. PQQ is also a powerful antioxidant protecting mitochondria against oxidative stress-induced lipid peroxidation, protein carbonyl formation and inactivation of the mitochondrial respiratory chain [10]. In addition to regulate mitochondrial biogenesis and mitigate oxidative stress-induced mitochondrial dysfunction, some studies indicated that PQQ could also enhance lipid β-oxidation, reduce lipid content and droplet size in animal liver and adipocytes [11, 12]. However, the beneficial effects and underlying mechanisms of PQQ during IVM on porcine oocyte maturation quality and their developmental competence have not been determined. Therefore, we investigated the effects of PQQ supplementation during porcine oocyte IVM on cumulus expansion, nuclear maturation, mitochondrial function, oxidative stress levels, lipid metabolism and developmental ability of embryos.

## MATERIALS AND METHODS

### Chemicals

All the chemicals and reagents used in this study were purchased from Sigma Aldrich (St. Louis, MO, USA) unless stated otherwise.

### Animal studies

The protocols for the animal studies were approved by and performed in accordance with the requirements of Ethics Committee of Medicine and Life Sciences of Inner Mongolia Minzu University (approval no. NMD-DW-2024-11-58).

### Oocyte collection and *in vitro* maturation

Porcine ovaries were obtained from a local slaughterhouse and transferred immediately to the laboratory in sterile 0.9% saline at 32°C to 35°C within 2 h. Follicular fluid containing cumulus oocyte complexes (COCs) was aspirated from about 3 to 6 mm antral follicles with an 18-gauge needle attached to a 10 mL disposable syringe. The COCs with compact cumulus cells and homogeneous cytoplasm of oocytes were collected and washed, and approximately 10 COCs were cultured in 50 μL of IVM medium covered with mineral oil for 42 h at 38.5°C with 5% CO2 in a humidified atmosphere. The IVM medium contained tissue culture medium 199 (TCM-199; Invitrogen, Waltham, MA, USA) supplemented with 10 IU /mL pregnant mare serum gonadotropin, 10 IU/mL human chorionic gonadotropin, 10 ng /mL epidermal growth factor, and 10% (v/v) porcine follicular fluid. During IVM, IVM medium was supplemented with different concentrations of PQQ (0 nM, 200 nM, 400 nM, 800 nM or 1,600 nM) depending on experimental design.

### Cumulus expansion assessment

**Figure 1:**
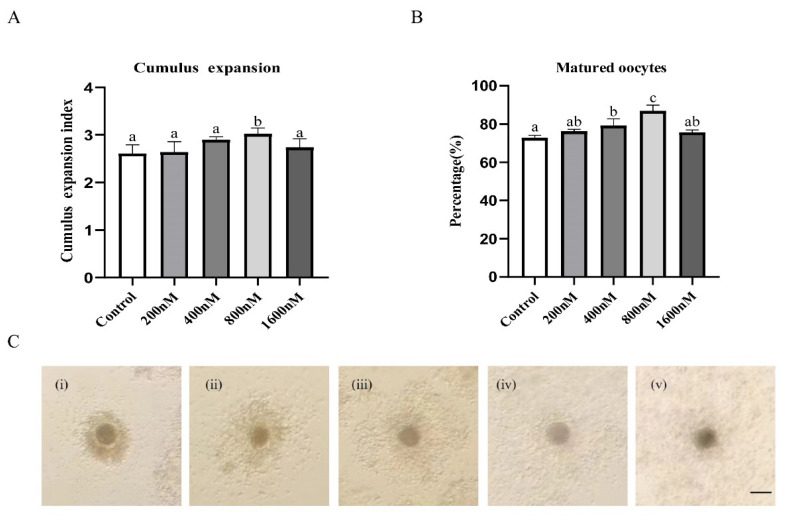
Effects of various concentrations of PQQ supplementation during IVM on cumulus expansion and oocyte nuclear maturation in porcine COCs. COCs were cultured for 42 h in IVM medium without or with PQQ (200 nM, 400 nM, 800 nM or 1,600 nM). Cumulus expansion index (CEI) and percentages of porcine oocytes reaching MII stage were detected after IVM. (A) CEI of COCs in control and PQQ-supplemented groups (n = 120–122/group). (B) The percentages of porcine oocytes reaching MII stage in control and PQQ-supplemented groups (n=120–122/group) (C) Morphological degrees of cumulus expansion in COCs after IVM, degree 0 (i), degree 1(ii), degree 2 (iii), degree 3 (iv), and degree 4 (v). Scale bars = 100 μm. Data are from at least four independent experiments and different superscript letters indicate signiﬁcant differences (p<0.05). PQQ, pyrroloquinoline quinone; IVM, in vitro maturation; COCs, cumulus oocyte complexes.

The degree of cumulus expansion was evaluated after IVM by microscopic examination. As shown in Figure 1C, grade 0: no expansion. Grade 1: minimum expansion, with spherical and compacted cumulus cells around the oocyte. Grade 2: outermost layer expansion of cumulus cells. Grade 3: the expansion of all layers of cumulus cells, except the corona radiata. Grade 4: complete expansion of all cumulus cell layers. Cumulus expansion index (CEI) was calculatedby by add up the scores of each COC (score 0 to 4) and dividing by the total number of COCs, as described previously [13].

### Evaluation of oocyte nuclear maturation

After IVM, oocytes were mechanically separated from surrounding cumulus cells by repeated pipetting. Denuded oocytes were fixed in 4% paraformaldehyde for 20 min and permeabilized with Triton X-100 (0.5%) for at least 30 min at room temperature, then stained with 10 μg/mL 4’,6-diamidino-2-phenylindole for 5 min. After washing, denuded oocytes were mounted on a sliding glass with a coverslip and examined under a fluorescence microscope (Eclipse Ci-s; Nikon, Tokyo, Japan). We measured oocyte maturation by the percentage of oocytes at metaphase II (MII) stage.

### Assessment of mitochondrial abundance and activity

Mitochondrial abundance and activity were evaluated with MitoTracker green (Molecular Probes, Eugene, OR, USA) and MitoTracker Red CMXRos (Beyotime, Shanghai, China), respectively. After IVM, oocytes were incubated with 200 nM MitoTracker green or 200 nM MitoTracker Red CMXRos in TCM199-based medium for 30 min at 37°C in the dark. Oocytes stained with MitoTracker green were washed three times in phosphate-buffered saline, and mounted and examined under a fluorescence microscope (Eclipse Ci-s; Nikon). Oocytes stained with MitoTracker Red CMXRos were washed three times in DPBS, and then fixed in 4% (w/v) paraformaldehyde for 15 minutes at 37°C. After fully washing, oocytes were mounted and examined under a fluorescence microscope (Eclipse Ci-s; Nikon). ImageJ software was used to analyze fluorescence intensity.

### Mitochondrial membrane potential measurement

Mitochondrial membrane potential (MMP) was detected using a MitoProbe JC-1 Assay kit (Beyotime) according to the manufacturer’s instructions. Briefly, denuded oocytes were incubated in culture medium at 37°C for 30 min with JC-1. After fully washing, the oocytes were mounted on glass slides and then imaged by the fluorescence microscope (Eclipse Ci-s; Nikon). ImageJ software was used to analyze fluorescence intensity. MMP was calculated as the ratio of red fluorescence intensity to green fluorescence intensity.

### Detection of intracellular reactive oxygen species and glutathione levels

Levels of ROS and glutathione (GSH) were measured as described by Wang et al [14]. Briefly, after IVM, matured COCs were denuded. Subsequently, denuded matured oocytes were stained for 30 min in the TCM199 medium containing 10 μM DCFH-DA or 10 μM CMF2HC at room temperature. After washing, oocytes were imaged by the fluorescence microscope (Eclipse Ci-s; Nikon). ImageJ software was used to analyze fluorescence intensity.

### Detection of lipid droplet and fatty acid contents

Lipid droplet and fatty acid analysis were performed using lipid droplets green fluorescence staining kit assay (BODIPY 493/503) and fatty acid green fluorescence probe (BODIPY 500/510 C1, C12), respectively. Briefly, after IVM, oocytes were ﬁxed in 4% paraformaldehyde for 15 min at room temperature. The fixed oocytes were washed three times in Dulbecco’s phosphate buffered saline (DPBS)-polyvinyl alcohol (PVA) and treated with BODIPY 493/503 (1:1000, Beyotime) or BODIPY 500/510 C1, C12 (6 μM, Beyotime) for 1 h at room temperature in the dark. After fully washing, oocytes were mounted on glass slides and then imaged by the fluorescence microscope (Eclipse Ci-s; Nikon). ImageJ software was used to analyze fluorescence intensity.

### Parthenogenetic activationand culture *in vitro*

After IVM, COCs were denuded with 0.1% hyaluronidase (w/v) by pipetting; then, parthenogenetic activation (PA) was induced with two direct-current pulses of 1.2 kV/cm for 60 μs in activation medium containing 0.3 M mannitol, 0.05 mM CaCl_2_·2H_2_O, 0.1 mM MgCl_2_·6H_2_O, and 0.5 mM 4- (2-Hydroxyethyl)-1-piperazineethanesulfonic acid and 0.1% bovine serum albumin (BSA) (w/v). The oocytes were washed three times in PZM-3 medium supplemented with 2 mM 6-dimethylaminopurine and 4 mg/mL BSA and incubated in the same medium for 4 h. Next, activated oocytes thoroughly washed and cultured in *in vitro* culture (IVC) medium for 7 days at 38.5°C in an atmosphere of 5% CO_2_ and 100% humidity. The day of PA was considered Day 0. Cleavage and blastocyst formation rate were evaluated on Day 2 and Day 7, respectively. Total cell number per blastocyst was assessed by 10 μg/mL Hoechst 33342 for 10 min to label the nuclei and washed three times with 1% PVA/DPBS (w/v), and then mounted onto glass slides. Images were captured by the fluorescence microscope (Eclipse Ci-s; Nikon).

### Quantitative real time polymerase chain reaction analysis

A CellAmpTM Direct SYBR RT-qPCR kit (Takara, Shiga, Japan) was used to produce cDNA according to the manufacturer’s instructions. Briefly, 50 oocytes of each group were lysed in Cell Lysis solution consisting of 48 μL Cell Lysis II Buffer and 2 μL DNase I at room temperature for 5 min. Reverse transcription was performed using a one-step procedure. Briefly, mixtures of each reaction containing 2 μL cell lysate, 4 μL 5× CellAmp Buffer II, 1 μL PrimeScript RT Enzyme Mix, 1 μL RT Primer Mix and 12 μL RNase Free H_2_O were added to a nuclease-free microfuge tube, and then incubated at 37°C for 30 minutes, and at 85°C for 5 seconds. Complementary DNA (cDNA) was stored at −20°C until used. The cDNA was quantified by qPCR using an ABI-Stepone Plus instrument (Applied Biosystems, Carlsbad, CA, USA). The amplification protocol included an initial denaturation step for 30 seconds at 95°C followed by 40 cycles consisting of denaturation for 5 seconds at 95°C, annealing for 15 seconds at 60°C. The 2^−ΔΔCt^ method was used to analyzed the gene expression level after the melting-curve analysis was completed. The expression levels of the target genes were then normalized to the expression level of *GAPDH* in each sample. The primer used were listed in Table 1. Each experiment was repeated independently three times.

### Statistical analysis

Each experiment was repeated at least three times, and experimental data are presented as means±standard error of the mean. Percentage data were subjected to arcsine transformation before statistical analysis. SPSS Statistics 18.0 was used for statistical analyses. To evaluate cumulus expansion and oocyte nuclear maturation, the one-way analysis of variance and Tukey’s test were utilized. All the other data were compared using the Student’s t-test. The differences were considered significant at p<0.05.

## RESULTS

### Effects of various concentrations of pyrroloquinoline quinone supplementation during *in vitro* maturation on cumulus expansion and oocyte nuclear maturation in porcine cumulus oocyte complexes

To evaluate the effect of PQQ on cumulus expansion and oocyte nuclear maturation, COCs were cultured in IVM medium supplemented with 0 nM (control), 200 nM, 400 nM, 800 nM or 1,600 nM PQQ. The results showed that 800 nM PQQ supplementation signiﬁcantly increased the CEI compared to the control and other PQQ treatment groups (Figures 1A, 1C; p<0.05). Supplementation with 800 nM PQQ during IVM also significantly increased oocyte maturation rate (Figures 1B, p<0.05). Therefore, 800 nM was selected as the optimum dose of PQQ, and subsequent experiments were conducted with the control and 800 nM PQQ groups.

### Effect of pyrroloquinoline quinone (800 nM) supplementation during *in vitro* maturation on mitochondrial function of porcine oocytes

**Figure 2:**
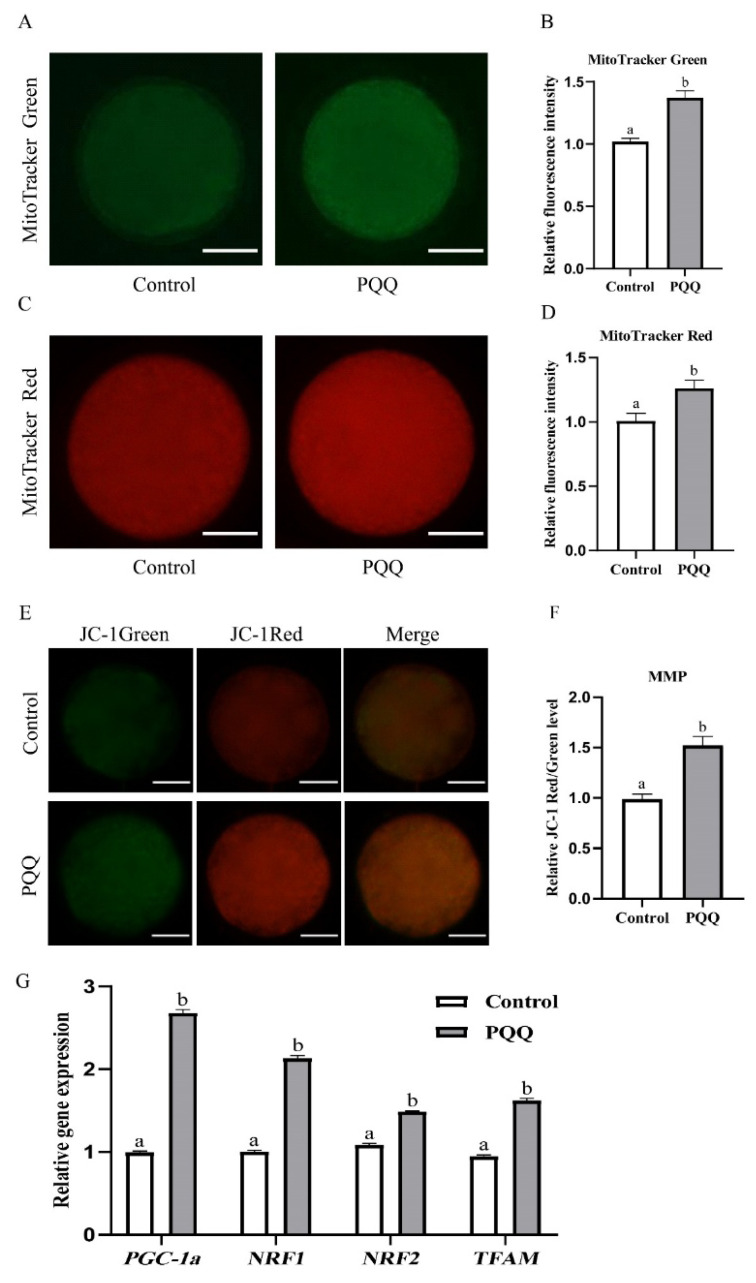
Effect of PQQ supplementation during IVM on mitochondrial function of porcine oocytes. COCs were cultured for 42 h in IVM medium without or with PQQ (800 nM). Mitochondrial abundance, mitochondrial activity, MMP levels, and transcripts abundance of genes related to mitochondrial biogenesis in oocytes were detected after IVM. (A) Representative images of oocytes stained with MitoTracker green in the control and PQQ-supplemented groups. (B) Fluorescence intensity of mitochondrial abundance in the control and PQQ-supplemented oocytes (Control, n = 96; PQQ, n = 88). (C) Representative images of oocytes stained with MitoTracker Red CMXRos in control and PQQ-supplemented groups. (D) Fluorescence intensity of mitochondrial activity in the control and PQQ-supplemented oocytes (Control, n = 68; PQQ, n = 60). (E) Representative images of oocytes stained with JC-1 in the control and PQQ-supplemented groups. (F) Relative MMP is represented as the ratio of red to green intensity in the control and PQQ-supplemented oocytes (Control, n = 64; PQQ, n = 64). (G) Relative mRNA transcripts abundance of mitochondrial biogenesis-related genes in the control and PQQ-supplemented oocytes (Control, n = 150; PQQ, n = 150). Scale bars = 50 μm. Data are from at least three independent experiments and different superscript letters indicate signiﬁcant differences (p<0.05). PQQ, pyrroloquinoline quinone; IVM, *in vitro* maturation; COCs, cumulus oocyte complexes; MMP, Mitochondrial membrane potential.

To determine the effects of PQQ on mitochondrial function, after IVM, we measured mitochondrial abundance, mitochondrial activity, MMP levels, and transcript abundance of genes related to mitochondrial biogenesis in porcine oocytes. The mitochondrial content, and mitochondrial activities and MMP were signiﬁcantly higher in PQQ-supplemented oocytes than in control oocytes (Figures 2A–2F; p<0.05). Furthermore, we also found that 800 nM PQQ supplementation signiﬁcantly increased mitochondrial biogenesis-related genes expression (*PGC-1a*, *NRF1*, *NRF2* and *TFAM*; Figure 2G; p<0.05).

### Effect of pyrroloquinoline quinone (800 nM) supplementation during *in vitro* maturation on oxidative stress levels of porcine oocytes

**Figure 3:**
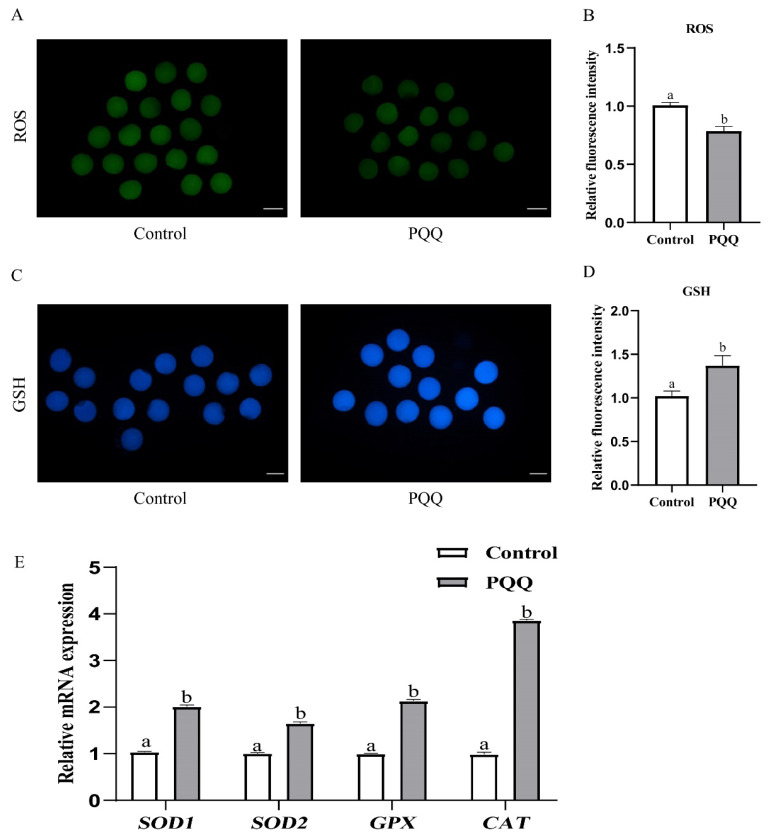
Effect of PQQ supplementation during IVM on oxidative stress levels of porcine oocytes. COCs were cultured for 42 h in IVM medium without or with PQQ (800 nM). Levels of ROS and GSH, and transcripts abundance of genes related to antioxidative stress in porcine oocytes were detected after IVM. (A) Representative images of porcine oocytes stained with DCFH-DA in the control and PQQ-supplemented groups. (B) Fluorescence intensity of ROS in the control and PQQ-supplemented oocytes (Control, n = 68; PQQ, n = 62). (C) Representative images of oocytes stained with CMF2HC in the control and PQQ-supplemented groups. (D) Fluorescence intensity of GSH in the control and PQQ-supplemented oocytes (Control, n = 72; PQQ, n = 65). (E) Relative mRNA transcripts abundance of oxidative stress-related genes in the control and PQQ-supplemented oocytes (Control, n = 150; PQQ, n = 150). Scale bars = 100 μm. Data are from at least three independent experiments and different superscript letters indicate signiﬁcant differences (p<0.05). PQQ, pyrroloquinoline quinone; IVM, *in vitro* maturation; COCs, cumulus oocyte complexes; ROS, reactive oxygen species; GSH, glutathione.

To examined the effect of PQQ on oxidative stress, after IVM, we measured ROS and GSH levels, and transcripts abundance of genes related to antioxidative stress in porcine oocytes. Results showed that 800 nM PQQ supplementation signiﬁcantly reduced ROS levels and increased GSH levels in oocytes compared to the control (Figures 3A–3D; p<0.05). Furthermore, we also found that 800 nM PQQ supplementation signiﬁcantly increased antioxidant-related genes expression (*SOD1*, *SOD2*, *GPX* and *CAT*; Figure 3E; p<0.05).

### Effect of pyrroloquinoline quinone (800 nM) supplementation during *in vitro* maturation on lipid metabolism of porcine oocytes

**Figure 4:**
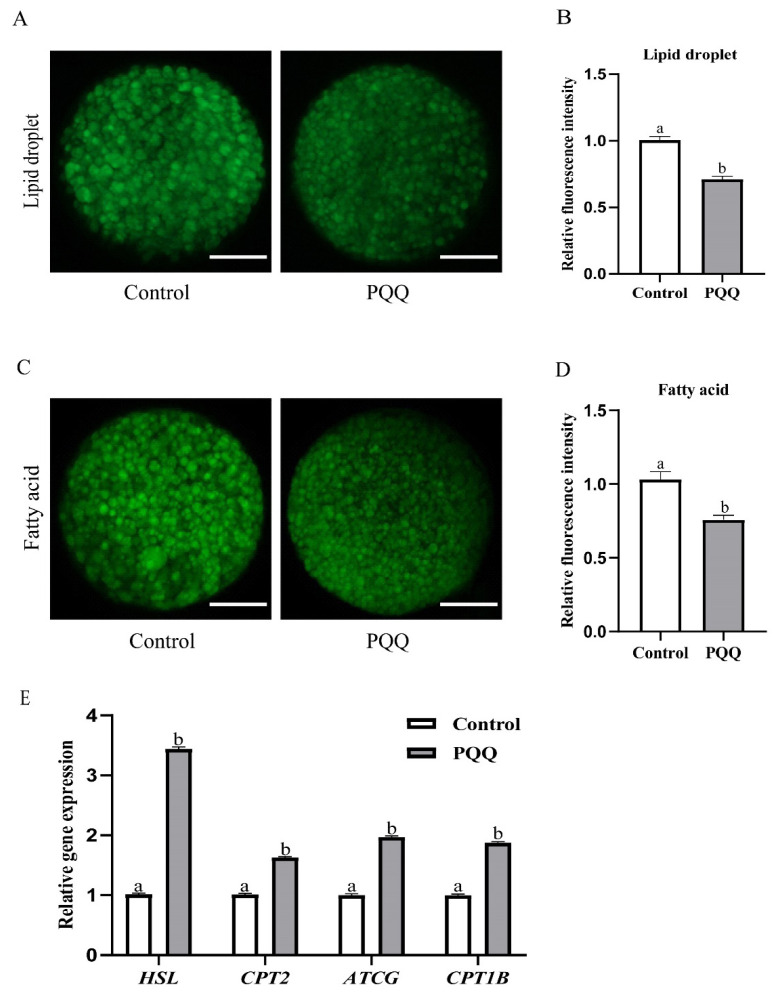
Effect of PQQ (800 nM) supplementation during IVM on lipid metabolism of porcine oocytes. COCs were cultured for 42 h in IVM medium without or with PQQ (800 nM). lipid droplets and fatty acids content, and transcripts abundance of genes-related to lipid metabolism in oocytes were detected after IVM. (A) Representative images of oocytes stained with BODIPY 493/503 in the control and PQQ-supplemented groups. (B) Fluorescence intensity of lipid droplets in the control and PQQ-supplemented oocytes (Control, n = 66; PQQ, n = 63). (C) Representative images of oocytes stained with BODIPY 500/510 C1, C12 in the control and PQQ-supplemented groups. (D) Fluorescence intensity of fatty acids in the control and PQQ-supplemented oocytes (Control, n = 60; PQQ, n = 62). (E) Relative mRNA transcripts abundance of lipid metabolism-related genes in the control and PQQ-supplemented oocytes (Control, n = 150; PQQ, n = 150). Scale bars = 50 μm. Data are from at least three independent experiments and different superscript letters indicate signiﬁcant differences (p<0.05). PQQ, pyrroloquinoline quinone; IVM, *in vitro* maturation.

To test whether PQQ affected lipid metabolism, after IVM, we detected lipid droplets and fatty acids content, and transcript abundance of genes related to lipid metabolism in porcine oocytes. Our results revealed that 800 nM PQQ supplementation signiﬁcantly decreased the fluorescence intensity of lipid droplets and fatty acids compared to the control (Figures 4A–4D; p<0.05). In addition, 800 nM PQQ supplementation signiﬁcantly upregulated lipolysis (*ATGL* and *HSL*) and β-oxidation (*CPT1B* and *CPT2*)-related mRNA expression levels compared to the control (Figure 4E, p<0.05).

### Effect of pyrroloquinoline quinone (800 nM) supplementation during *in vitro* maturation on the developmental competence of porcine parthenogenetic activation embryos

**Figure 5:**
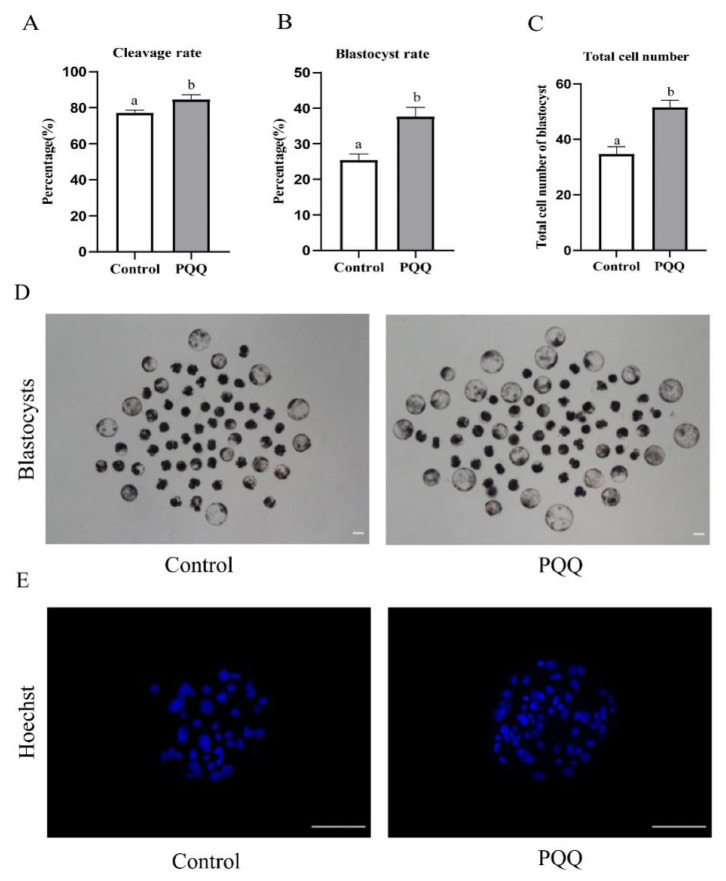
Effect of PQQ supplementation during IVM on the developmental competence of porcine PA embryos. COCs were cultured for 42 h in IVM medium without or with PQQ (800 nM). Subsequently, oocytes were parthenogenetically activated to evaluate cleavage rate, blastocyst formation rate, and the total cell numbers in the blastocyst after IVC for 7 d. (A) Cleavage rates in control and PQQ-supplemented groups on day 2 (Control, n = 265; PQQ, n = 285). (B) Blastocyst formation rates in the control and PQQ-supplemented groups on day 7 (Control, n = 265; PQQ, n = 285). (C) Total number cell of blastocysts in control and PQQ-supplemented groups on day 7 (Control, n = 45; PQQ, n = 58). (D) Representative images of the embryos at blastocyst stage in the control and PQQ-supplemented groups. Scale bars = 100 μm. (E) Representative images of blastocysts stained with Hoechst 33342 in the control and PQQ-supplemented groups. Scale bars = 100 μm. Data are from at least four independent experiments and different superscript letters indicate signiﬁcant differences (p<0.05). PQQ, pyrroloquinoline quinone; IVM, *in vitro* maturation; COCs, cumulus oocyte complexes.

To further evaluate the oocyte developmental capacity, after IVM, PA was performed, cleavage rate, blastocyst formation rates, and the total cell numbers in the blastocyst were then examined. Results demonstrated that cleavage rate, blastocyst rates, and the total cell numbers in the blastocyst were significantly higher in the 800 nM PQQ-treated group than that in the control group (Figures 5A–5E; p<0.05).

## DISCUSSION

The present study investigated whether the effects of PQQ supplementation during IVM on porcine oocyte maturation and embryo developmental competence. We found that adding PQQ to an IVM medium significantly improved cumulus expansion, oocyte maturation rate, cleavage rate, blastocyst rate, and the total cell numbers in the blastocyst after partheno-genetic activation. At the same time, the expressions of genes related to antioxidant, mitochondrial biogenesis and lipid metabolism in porcine oocyte were significantly increased. Moreover, PQQ treatment decreased oxidative stress, lipid droplets and fatty acids content, improved mitochondrial abundance, metabolism activity and membrane potential. These results indicated PQQ can positively influence porcine oocyte maturation and regulate mitochondrial function and lipid metabolism.

Cumulus expansion degree and nuclear maturation rate are considered as the key indicators of oocyte maturation quality. Our data showed that PQQ supplementation elevated the rate of MII oocytes and CEI, indicating the beneficial effects of PQQ on the oocyte maturation. Although previous studies have shown that antioxidants, such as quercetin, cysteamine, carnitine, vitamin C, resveratrol or Tannin, may improve CEI and promote cytoplasmic maturation, these antioxidants, unlike PQQ, have no signiﬁcant effects on nuclear maturation rate and the total cell numbers in the blastocyst [15, 16].

Mitochondrion is an important organelle located in the cytoplasm and the functional state of the mitochondria is critical for providing energy to drive the normal oocyte development [17]. The number of mitochondria, mitochondrial membrane potential and its metabolism activity are closely associated with increased oocyte quality [18–20]. Our results showed that PQQ supplementation during IVM improved mitochondrial abundance, mitochondrial activity and MMP. Furthermore, we also found that PQQ upregulated mitochondrial biogenesis-related transcript expressions (*PGC-1α*, *NRF1*, *NRF2* and *TFAM*). PGC-1α is known to be a central transcriptional coactivator inducing mitochondrial biogenesis. NRF1 and NRF2 regulate expression of the nuclear-encoded respiratory chain components and TFAM. TFAM is major regulator of mitochondrial DNA transcription and replication. Numerous studies have shown that the increased expression of *PGC-1α* enhanced mitochondrial biogenesis by modulating the gene expression of *NRF1*, *NRF2* and *TFAM*, resulting in improved mitochondrial function [21–23]. Similarly, previous studies showed that some bioactive substances (Mogroside V, notoginsenoside R1, Nobiletin) supplementation to IVM medium promoted mitochondrial biogenesis and increased mitochondrial activity and MMP in porcine oocytes [24–26]. Thus, our data demonstrated that PQQ exerted a beneficial effect on oocyte maturation and subsequent embryonic development by modulating the mitochondrial biogenesis and function.

It is believed that IVC conditions induce excessive generation of ROS in oocytes due to the exposure to environmental stressors. Excessive generation of ROS will cause DNA fragmentation, spindle abnormalities, mitochondrial dysfunction and apoptosis, ultimately impairing oocyte maturation quality and reducing embryo development [27, 28]. Notably, PQQ supplementation significantly reduced ROS levels in porcine oocyte. This may have been due to the fact that PQQ upregulated the expression levels of genes related to antioxidant stress (*SOD1*, *SOD2*, *GPX* and *CAT*). SOD, GPX and CAT are key enzymatic antioxidants within cells, which convert oxidants into compounds that are harmless to the cell. Moreover, PQQ supplementation also increased the levels of GSH, while decreasing intracellular ROS levels. These results are similar to those of some previous reports using antioxidants, like resveratrol, melatonin or luteolin supplementation to IVM medium [29–31]. GSH is a key marker to evaluate the cytoplasmic maturation of oocytes [32]. Increased levels of intracellular GSH can scavenge ROS and increase mitochondrial activity, thereby improving oocyte maturation quality and their developmental capacity [33]. Furthermore, some studies have showed that antioxidants supplementation during oocytes IVM can prevent mitochondrial dysfunction by reducing ROS levels [34, 35]. Importantly, in this study, PQQ-supplemented oocytes not only reduced ROS levels, but also increased GSH levels, mitochondrial abundance, MMP and mitochondrial activity compared to the control, indicating that PQQ supplementation improved mitochondrial content and function partly due to its antioxidant activity. Our findings suggest that PQQ promotes porcine oocyte maturation and early embryonic development by mitigating oxidative stress.

It has been proven that pig oocytes contain large quantities of fatty acids that are stored as neutral lipids in the form of lipid droplets [36]. Fatty acids are generated by lipolysis and transported into the mitochondria, where they are further utilized by β-oxidation for energy production [37]. Previous studies showed that inhibition of fatty acids β-oxidation during IVM decreases the developmental competence of oocyte in both laboratory and livestock species [38, 39], whereas stimulation of fatty acids β-oxidation during IVM enhances the developmental competence of oocyte [40]. These results indicate that fatty acids β-oxidation during oocyte IVM is essential for oocyte maturation and subsequent embryo development. HSL and ATGL are key enzymes that modulate triglycerides degradation within cells. ATGL catalyzes triglycerides hydrolysis to diglycerides and free fatty acids, and HSL subsequently hydrolyzes diacylglycerols and produces monoacylglycerol and free fatty acids. CPT1B and CPT2 are key enzymes that maintain the transport of fatty acids into the mitochondria to β-oxidation. More importantly, this study showed that PQQ supplementation during IVM significantly reduced the levels of lipid droplets and fatty acids in porcine oocytes and increased the expression levels of genes related to lipolysis (*HSL*, *ATGL*) and β-oxidation (*CPT1B*, *CPT2*). These results demonstrate the beneﬁcial effects of PQQ on cytoplasmic maturation of porcine oocytes through enhancement of lipid metabolism. Although some bioactive substances, such as Mogroside V, notoginsenoside R1 and Nobiletin, may promote mitochondrial function in porcine oocytes, these bioactive substances, unlike PQQ, have not been shown definitively to have beneficial effects on lipid metabolism [24–26].

## CONCLUSION

Our findings demonstrate that the addition of PQQ to IVM medium is an effective approach to improve oocyte maturation quality and subsequent embryo development by alleviating oxidative stress and enhancing mitochondrial function and lipid metabolism. In addition, this study helps raise awareness of the beneﬁcial effects of PQQ on oocyte maturation and early embryo development using a porcine model that may offer a potential strategy for treating female infertility.

## Figures and Tables

**Table 1:** Primer sequences used for real-time PCR

Gene	Primer sequences	GenBank accession no.	Product size (bp)
*SOD1*	F: GTTGGAGACCTGGGCAATGT R: CGGCCAATGATGGAATGGTC	NM_001190422.1	104
*SOD2*	F: CCCTGGAGCCGCACATC R: TTTTTCAGCGCCTCCTG	NM_214127.2	115
*CAT*	F: ATGTGCAGGCTGGATCTCAC R: GCACAGGAGAATCTTGCATCC	XM_021081498.1	155
*GPX*	F: GAAGTGTGAGGTGAATGGCG R: CTCGAAGTTCCATGCGATGT	NM_214201.1	157
*PGC-1a*	F: GCCCTCATTTGATGCACTG R: AGCTGAGTGTTGGCTGGTG	XM_021100444.1	150
*TFAM*	F: TGCTTTGTCTACGGGTGCAA R: ACTTCCACAAACCGCACAGA	NM_001130211.1	100
*NRF1*	F: ACCATCCAGACAACGCAA R: ACTCCAGTAAGTGCTCCGAC	XM_021079000.1	230
*NRF2*	F: GCCCAGTCTTCATTGCTCCT R: AGCTCCTCCCAAACTTGCTC	XM_021075133.1	115
*HSL*	F: TGTCTTTGCGGGTATTCG R: TTGTGCGGAAGAAGATGC	NM_214315.3	209
*ATGL*	F: CGAACTCAAGAGCACCATCA R: TTGCACATCTCTCGAAGCAC	XM_021076533.1	189
*CPT2*	F: AGTTCCAGAGAGGAGGCAAAG R: GAGCATCTCTTGGTGAAGACG	NM_001246243.1	199
*CPT1B*	F: ATCAAGCCTGTGATGGCTCT R: GAGCCACACCTTGAAGAAGC	XM_021091195.1	168
*GAPDH*	F: GTCGGTTGTGGATCTGACCT R: TTGACGAAGTGGTCGTTGAG	NM_001206359.1	207

PCR, polymerase chain reaction; F, forward; R, reverse; *SOD1*, superoxide dismutase 1; *SOD2*, superoxide dismutase 2; *CAT*, Catalase; *GPX*, glutathione peroxidase; *PGC-1a*, peroxisome proliferator-activated receptor-γ coactivator-1α; *TFAM*, mitochondrial transcription factor A; *NRF1*, nuclear respiratory factor 1; *NRF2*, nuclear respiratory factor 2; *HSL*, hormone-sensitive lipase; *ATGL*, adipose triglyceride lipase; *CPT2*, carnitine palmitoyltransferase 2; *CPT1B*, carnitine palmitoyltransferase 1B; *GAPDH*, glyceraldehyde 3-phosphate dehydrogenase.
